# Supplementary material for: Myeloperoxidase promotes fibrosis by inhibiting cathepsin K to bias the lung toward ECM accumulation
Source: bioRxiv. 2026 Apr 7:2026.04.05.713467. Preprint. [Version 1] doi: 10.64898/2026.04.05.713467 (PMC13082008; doi:10.64898/2026.04.05.713467)
Supplement: Supplement 1 [file media-1.pdf]

Supplemental figure 1. Flow sorting strategy.

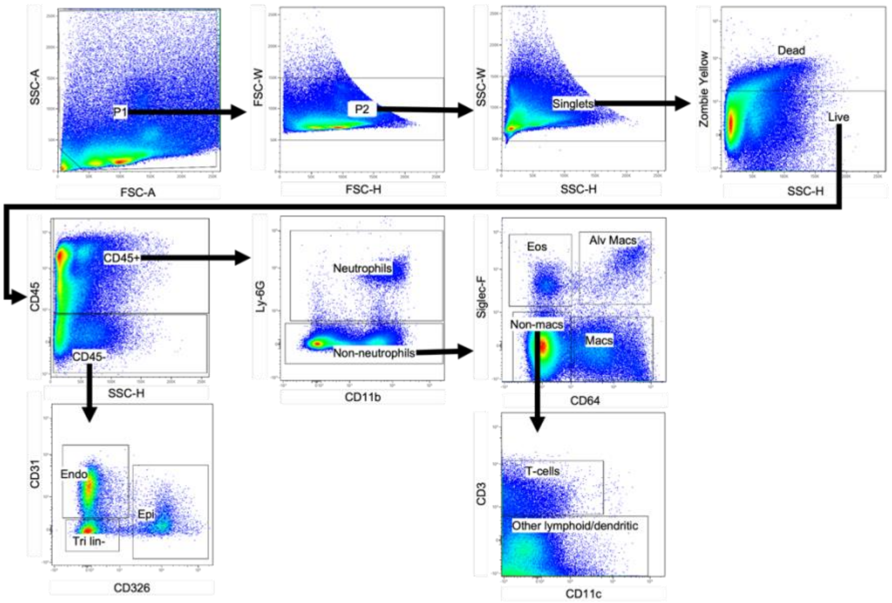

Supplemental figure 2. Fluorescent probe of cathepsin k activity does not increase in response to H2O2 or MPO.

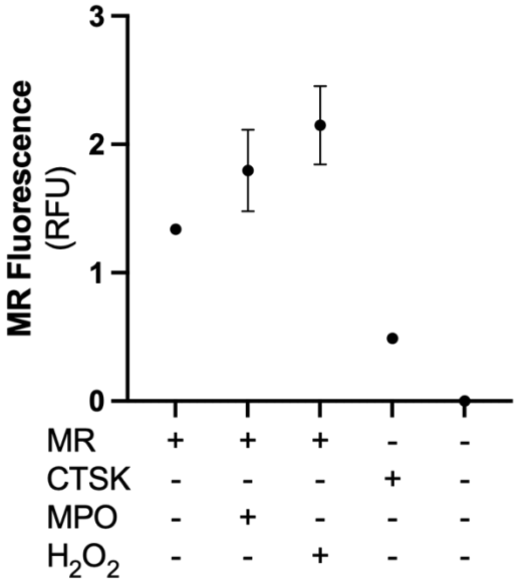

Supplemental Figure 3. Quantification of fibrotic area analysis from MTC (A) and lung injury from H&E staining (B).

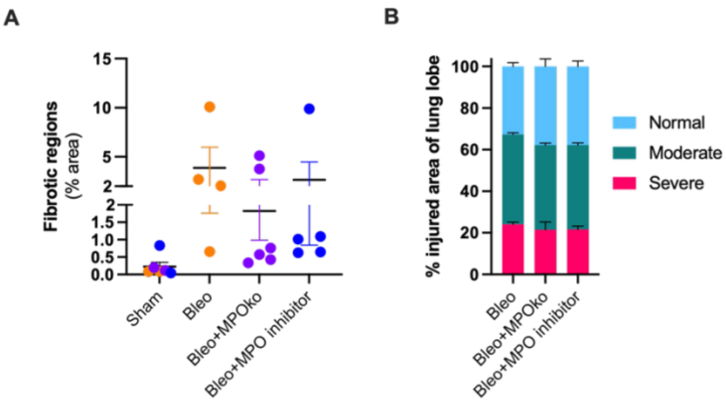

Supplemental figure 4. IVIS and homogenized lung tissue with cathepsin k fluorescent activity probe demonstrate increased cathepsin k activity in MPOko mice after bleomycin.

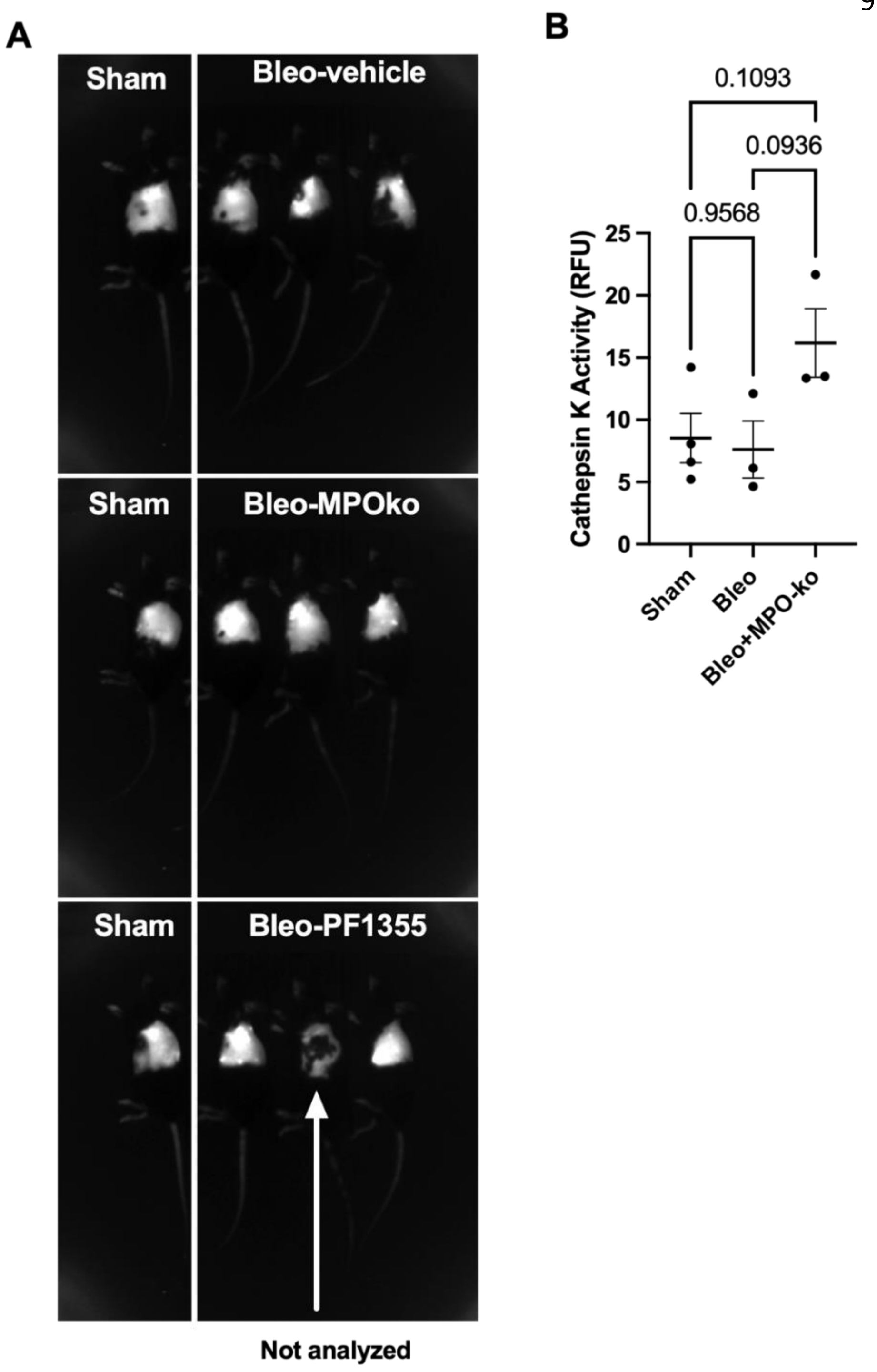

Supplement figure 5. Frequencies of immune cells are not different between MPOko and WT animals after bleomycin.

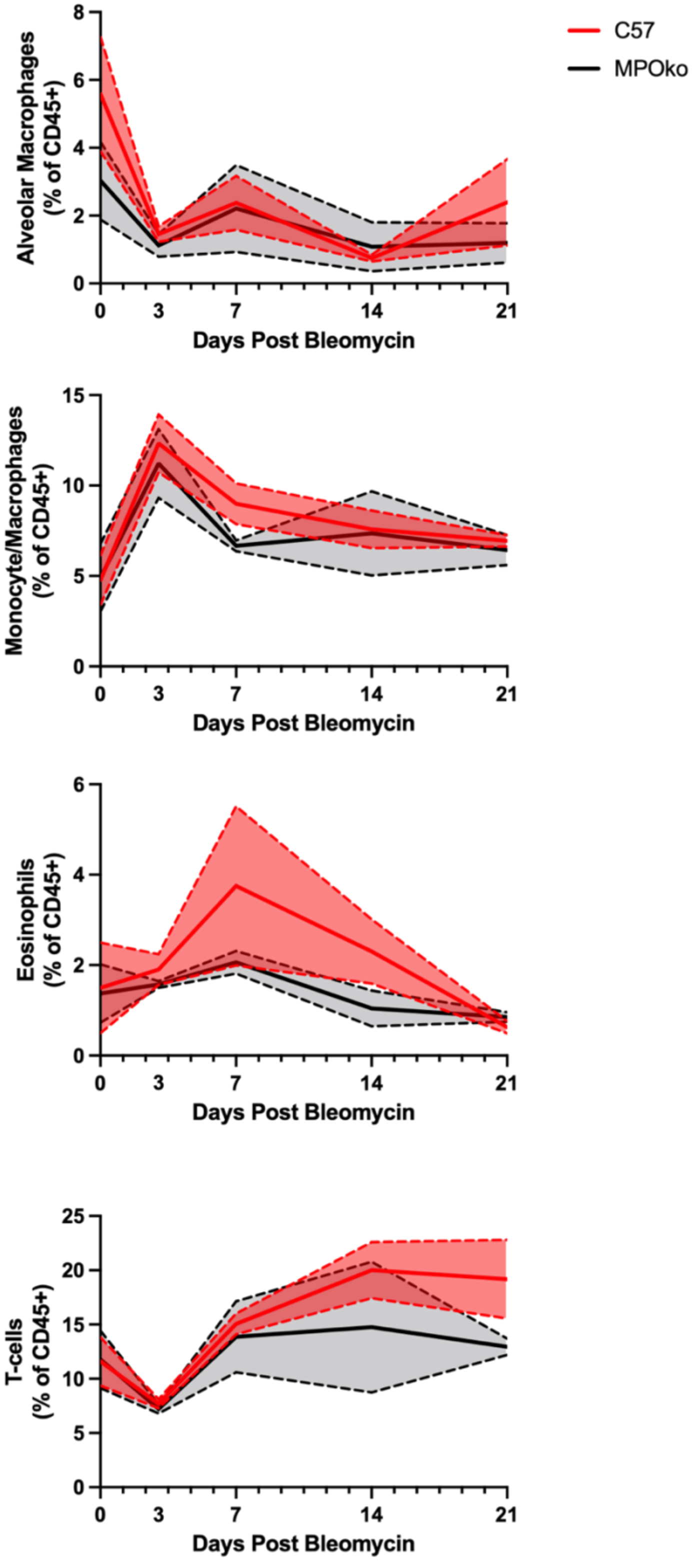

Supplemental Table 1. Patient demographics for samples used in this study.

Patient Demographics					
**Characteristics**		**IPF**		**Healthy**	
Median age (Range)		67 (47-86)		65 (47-82)	
Gender					
	M	18	67%	9	56%
	F	9	33%	7	44%
CT pattern					
	Probable UIP	12	44%	-	-
	UIP	6	22%	-	-
	Indeterminate	1	4%	-	-
	Unknown	8	30%	-	-
Antifibrotic				-	-
	Yes	6	22%	-	-
	No	13	48%	-	-
	Unknown	8	30%	-	-

Supplemental table 2. Reagents used in this study.

Supplemental Table 2. Equipment and Products Used in These Studies					
**Category**	**Product**	**Product Number**	**RRID**	**Vendor**	Model/Version/Lot
**Reagents**	Hospira Bleomycin	0409-0332-20	—	Vizient Inc.	BL12303B
	20 ga catheter, Angiocath	26742	—	BD	
	PBS	14190250	—	Life Technologies	
	PF-1355 MPO inhibitor	HY-100873	—	MedChemExpress	
	Isoflurane		—		
	Ketamine		—		
	Xylazine		—		
	Liberase TM	5401127001	—	Sigma-Aldrich	
	DNase I	4536282001	—	Sigma-Aldrich	
	40 µm filter	SCNY00040	—	Sigma-Aldrich	
	RBC Lysis buffer	420301	—	BioLegend	
	30 µm filter	NC9682496	—	Sysmex America Inc.	
	AutoMACS Buffer	130-091-222	—	Miltenyi Biotec	
	Eagle's Minimum Essential Medium (EMEM)	30-2003	—	ATCC	
	Alveolar Epithelial Medium Complete Kit (AEpiCM)	3201	—	Sciencell	
	DMEM	11965118	—	Life Technologies	
	Anti-Anti	15-240-062	—	Fisher Scientific	
	FBS	30-2020	—	ATCC	
	Trypsin-EDTA Solution 0.25% Cell Culture Tested	T4049-100ML	—	Sigma-Aldrich	
	Alexa Fluor® 700 anti-mouse CD3 Antibody	100216	AB_493697	BioLegend	
	PerCP/Cyanine5.5 anti-mouse CD11c Antibody	117328	AB_2129641	BioLegend	
	APC/Cyanine7 anti-mouse/human CD11b Antibody	101226	AB_830642	BioLegend	
	PE anti-mouse CD64 (FcγRI) Antibody	161003	AB_2904306	BioLegend	
	PE/Cyanine7 anti-mouse Ly-6G Antibody	127618	AB_1877261	BioLegend	

	Brilliant Violet 421™ anti-mouse CD170 (Siglec-F) Antibody	155509	AB_2810421	BioLegend	
	Zombie Yellow™ Fixable Viability Kit	423104	—	BioLegend	
	BV711 anti-mouse CD45 Antibody	103147	AB_2564383	BioLegend	
	Brilliant Violet 785™ anti-mouse CD31 Antibody	102435	AB_2810334	BioLegend	
	Brilliant Violet 650™ anti-mouse Ly-6C Antibody	128049	AB_2800630	BioLegend	
	FITC anti-mouse CD326 (Ep-CAM) Antibody	118208	AB_1134107	BioLegend	
	Purified Anti-Mouse CD16 / CD32 (Fc Shield) (2.4G2)	70-0161-U500	AB_2621487	Cytek	
	True-Stain Monocyte Blocker	426102	—	BioLegend	
	Masson's Trichrome Stain	25088-1	—	Polysciences Inc.	
	Hematoxylin Solution, Gill No. 3	GHS332-1L	—	Sigma-Aldrich	
	Eosin Y	E4009	—	Sigma-Aldrich	
	Picro-Sirius Red Solution	ab246832	—	Abcam Inc.	
	To-Pro-3	T3605	—	ThermoFisher	
	Hoechst 33342, trihydrochloride trihydrate	H1399	—	ThermoFisher	
	Alexa 555 anti-goat secondary antibody	62248	—	ThermoFisher	
	Hydroxyproline Colorimetric Assay Kit	K555-100	—	BioVision	
	PINP ELISA kit: Mouse PINP ELISA Kit	MBS2500076	—	MyBioSource	
	Human Myeloperoxidase ELISA Kit - Quantikine	DMYE00B	—	R&D Systems	

	CTXI ELISA kit: Mouse CTXI (Cross Linked C-Telopeptide of Type I Collagen) ELISA Kit	MBS9141384	—	MyBioSource	
	MPO ELISA DuoSet anti-Mouse	DY3667	—	R&D Systems	
	UltraPure™ Low Melting Point Agarose	16520100	—	ThermoFisher	
	Gelatin from porcine skin, powder, gel strength ~300 g Bloom, Type A, BioReagent, for electrophoresis, suitable for cell culture	G1890-100G	—	Sigma-Aldrich	
	CaCl2	C4901-500G	—	Sigma-Aldrich	
	MgSO4	M7506-500G	—	Sigma-Aldrich	
	Magic Red	940	—	ImmunoChemistry	
	recombinant active MPO	3174-MP-250	—	R&D	
	MPO isolated from human leukocytes	475911	—	Sigma-Aldrich	
	MPO isolated from human leukocytes	M6908	—	Sigma-Aldrich	
	Recombinant Active Human Cathepsin K	ab157067	—	Abcam	
	In vivo cathepsin K activity reporter	NEV11000	—	Perkin Elmer	
	anti-MPO antibody	AF3667	AB_2250866	R&D Systems	
	4-Aminobenzoic Acid hydrazide (ABAH)	14845	—	Cayman Chemical	
	Human TGF-beta 1 Recombinant Protein, PeproTech®	100-21C-10UG	—	ThermoFisher	
	Hydrogen Peroxide	16911-250ML-F	—	Sigma-Aldrich	
	EDTA, 0.5M Solution 100ml	C001N18 - 4055-100ML	—	Thomas Scientific	
	T-PER™ Tissue Protein Extraction Reagent	78510	—	ThermoFisher	

	HBSS (10x), no calcium, no magnesium, no phenol red	14185052	—	ThermoFisher	
	Sodium Acetate, 3M, pH 5.2, Molecular Biology Grade - CAS 127-09-3 - Calbiochem	567422	—	Sigma-Aldrich	
	L-Ascorbic Acid (White Crystalline Powder), Fisher BioReagents	BP351-500	—	Fisher Scientific	
**Organisms**	IMR-90 (human lung fibroblast)	CCL-186	CVCL_0347	ATCC	
	MPO-ko mice (B6.129X1-Mpotm1Lus/J)	004265	IMSR_JAX:004265	Jackson Labs	
	C57BL/6J mice	000664	IMSR_JAX:000664	Jackson Labs	
**Equipment**	LSR Flow Cytometer	Fortessa X-20	—	BD Biosciences	
	Fluorescent Plate Reader	FlexStation 3	—	Molecular Devices	
	Brightfield Slide Scanner	EasyScan Pro	—	Motic	
	Axio Observer Widefield Fluorescent Microscope	Axio Observer	—	Ziess	
	Tissue Homogenizer	BeadMill 24	—	ThermoFisher	
	Axioscan slide scanner	AxioScan	—	Zeiss	
	IVIS Spectrum In Vivo Imaging Systems	IVIS Spectrum	—	Perkin Elmer	
	Microscope (Confocal)	CKX53	—	Olympus	
**Software**	EasyScan		SCR_024854	Motic	
	Statistical Analysis Software		SCR_002798	GraphPad Prism	v10.6
	Image Analysis Software		SCR_002285	ImageJ (Fiji)	v1.53t
	Flow Cytometry Analysis Software		SCR_008520	FlowJo	v10.8
	Fluorescent Plate Reader Software		SCR_014240	Molecular Devices SoftMax Pro	v7
